# The gut‐microbiota‐brain axis: Focus on gut steroids

**DOI:** 10.1111/jne.13471

**Published:** 2024-11-22

**Authors:** Silvia Diviccaro, Silvia Giatti, Lucia Cioffi, Gabriela Chrostek, Roberto Cosimo Melcangi

**Affiliations:** ^1^ Department of Pharmacological and Biomolecular Sciences University of Milan Milan Italy

**Keywords:** allopregnanolone, PFS, pregnenolone, PSSD, SSRIs

## Abstract

There are over 1000 varieties of steroids that have been reported in nature, including the endogenous sex steroid hormones (i.e., progesterone, testosterone, and 17β‐estradiol) and corticosteroids which are mainly synthesized by gonads and adrenals, respectively. In addition, an extra‐glandular steroidogenesis has been also reported in the brain and in the gastrointestinal tract (GIT). The reason why intestinal steroidogenesis and consequently gut steroids draw our attention is for the communication and interaction with the gut microbiota, which functions like a virtual endocrine organ, and it is also involved in the steroid production. Moreover, both GIT and gut microbiota communicate through neural, endocrine, and humoral ways with the brain, in the so‐called gut‐microbiota‐brain axis. On this basis, in this review, we will discuss several aspects such as (1) intestinal steroidogenesis and its possible regulation, (2) the potential role of gut steroids in physiopathological conditions, and (3) the role of microbiome in steroidogenesis and steroid metabolism. Overall, this review highlights new points of view considering steroid molecules as potential therapeutic approach for gastrointestinal disorders and brain comorbidities.

## 1 INTRODUCTION

It has been 100 years since the word “hormone” was first introduced. The term, derived from the Greek participle ὁρμῶν, meaning “to arouse or excite,” was coined by Ernest H. Starling to describe the chemical regulation of the bodily functions.^1^ Numerous molecules are involved in these chemical pathways that regulate physiology and behavior, including steroids. Classic endocrinology identifies peripheral steroidogenic glands, such as gonads and adrenals, as the primary sites that synthesize and release steroid hormones into the bloodstream, where they act at distant target sites. However, local steroid production, known as extra‐glandular steroidogenesis, also occurs in other tissues and organs, including the thymus,^2^ skin,^3^ lung,^4^ and both the central and peripheral nervous system.^5^ This review focuses on the gastrointestinal tract (GIT) as another site of local steroid production. Indeed, de novo synthesis and metabolism of steroids in animal models have been reported.^6^, ^7^, ^8^, ^9^, ^10^, ^11^, ^12^, ^13^, ^14^ All steroid molecules synthesized in the gut are referred as “gut steroids.”

It is plausible that while classical peripheral glands use endocrine, paracrine, and autocrine mechanisms, extra‐glandular tissues primarily rely on paracrine‐ or autocrine‐like pathways, suggesting that local synthesis is necessary to meet in‐situ demands. Endocrine, paracrine, and autocrine effects are mediated by both classical steroid receptors (nuclear steroid receptors acting as transcriptional factors) and non‐classical steroid receptors (mainly membrane receptors, able to alter membrane potential and to start intracellular signaling).^15^, ^16^, ^17^, ^18^, ^19^, ^20^, ^21^, ^22^, ^23^, ^24^ Interestingly, both receptor classes are expressed in the GIT,^25^, ^26^, ^27^, ^28^ underscoring the importance of steroid molecules in gut physiology. Notably, the local production is constantly in harmony with the bloodstream, maintaining a balance between peripheral and brain environments, in a process called homeostasis.

Over the past decade, the rapidly expanding field of the gut‐microbiota‐brain axis (GMBA) has broadened the horizons of endocrinology. The GMBA refers to the complex, bidirectional communication network that links the gut microbiota (the trillions of microorganisms living in the GIT) and the brain. This system connects the central nervous system (CNS) with the enteric nervous system (ENS), influencing not only digestive processes but also brain function and behavior.^29^ It is considered a key player in the interaction between mental health, cognitive function, and physical well‐being.^30^ This development has enhanced scientific research and potentially improved therapeutic approaches, aligning with the goals of personalized medicine.^31^, ^32^ In light of new scientific evidence that highlights the interaction between the gut and brain, it is important to assess how steroids may influence the gut microbiome and, conversely, how the gut microbiome may impact steroid production, and ultimately, the brain's response. Although significant progress has been made, many questions remain unanswered. In this review, we will address key aspects of the potential role of gut steroids in the GMBA, both in physiological and pathological conditions.

## 2 INTESTINAL STEROIDOGENESIS AND GUT STEROIDS

**FIGURE 1:**
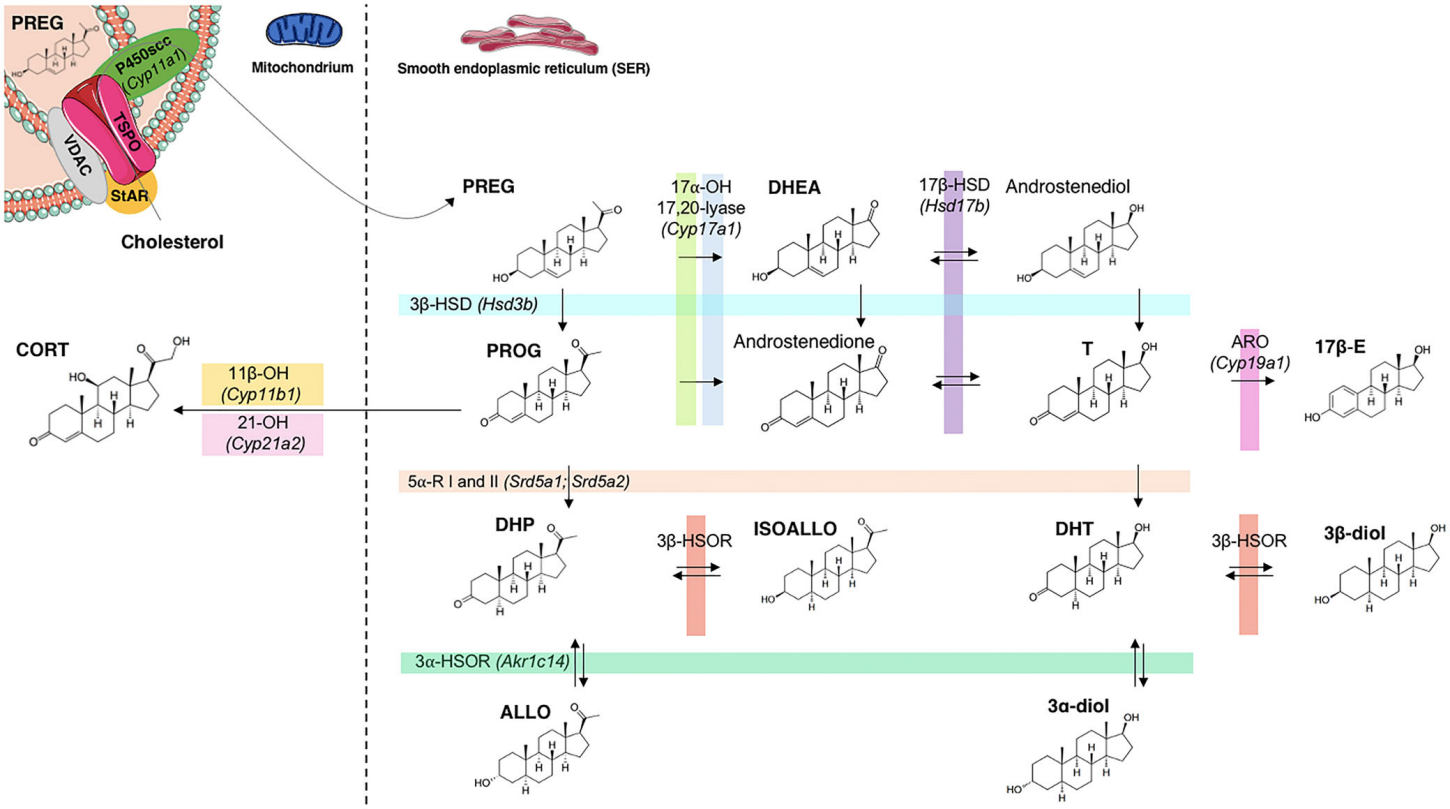
Intestinal steroidogenesis. Schematic diagram showing the putative pathway for steroidogenesis in the rodent intestine between mitochondria and the smooth endoplasmic reticulum. The genes related to each enzyme of *Rattus norvegicus* are reported in brackets. The arrows and bidirectional arrows indicate the irreversible and reversible reactions respectively. 11β‐OH/*Cyp11b1*, 11β‐hydroxylase; 21‐OH/*Cyp21a2*, 21‐hydroxylase; 3α‐HSOR/*Akr1c14*, 3α‐hydroxysteroid oxidoreductase; 3β‐HSD/*Hsd3b*, 3β‐hydroxysteroid dehydrogenase; 3β‐HSOR, 3β‐hydroxysteroid oxidoreductase; 5α‐R1/*Srd5a1*, 5α‐reductase type 1; 5α‐R2/*Srd5a2*, 5α‐reductase type 2; ARO/*Cyp19a1*, aromatase; *Cyp17a1*, P450 CYP17; StAR, steroidogenic acute regulatory protein; TSPO, translocator protein of 18 kDa; VDAC, voltage‐dependent anion channels. For the other abbreviations, see the text.

Is well‐known that the steroid synthesis (i.e., steroidogenesis) uses cholesterol as a substrate in a series of biochemical reactions occurring in the mitochondria and smooth endoplasmic reticulum (Figure 1). The first and rate‐limiting step of steroidogenesis is the translocation of free cholesterol into the mitochondria, a process primarily mediated by the steroidogenic acute regulatory protein, which collaborates with a protein complex including voltage‐dependent anion channels and translocator protein of 18 kDa.^33^, ^34^ Once inside the mitochondria, cholesterol interacts with the P450 side‐chain cleavage enzyme (P450scc; *Cyp11a1*), which converts it into pregnenolone (PREG). PREG then diffuses into the cytosol, where it undergoes further metabolism into various active steroids, as depicted in Figure 1.

Other key enzymes in the steroidogenesis include 3β‐hydroxysteroid dehydrogenase (3β‐HSD), which converts PREG into progesterone (PROG), and the P450 CYP17 enzyme complex, which includes 17α‐hydroxylase and 17,20‐lyase. These enzymes convert PREG into dehydroepiandrosterone (DHEA), which is subsequently metabolized into testosterone (T) by 17β‐hydroxysteroid dehydrogenase (17β‐HSD). Additionally, 5α‐reductase type 1 and type 2, convert PROG and T into dihydroprogesterone (DHP) and dihydrotestosterone (DHT), respectively.

Moreover, 3α‐ or 3β‐hydroxysteroid oxidoreductase (3α‐HSOR or 3β‐HSOR) enzymes are also involved in synthesizing active metabolites of DHP, such as allopregnanolone (ALLO) and isoallopregnanolone (ISOALLO), as well as metabolites of DHT, including 5α‐androstane‐3α, 17β‐diol (3α‐diol) and 5α‐androstane‐3β, 17β‐diol (3β‐diol). Additionally, aromatase converts T into 17β‐estradiol (17β‐E).

With regard to glucocorticoids, corticosterone (CORT) and cortisol (depending on the species) are synthesized from PROG through the cooperation of two enzymes: 21‐hydroxylase and 11β‐hydroxylase. However, to date, there is no evidence of the synthesis of another corticosteroids (e.g., mineralocorticoids) in the intestine.

All the steps described above have been observed in animal models as evidence of local intestinal steroidogenesis.

Several studies have focused on the enzymes involved in glucocorticoids—known for their anti‐inflammatory properties—in the small and large intestines. These studies provide the first indications of interactions between gut steroids and immune system.^7^ Notably, *Cyp11a1* and *Cyp11b1* mRNA are expressed both in a murine intestinal epithelial cell line^35^ and in human colon biopsies,^36^ underscoring the importance of investigating steroid metabolism beyond to glucocorticoids, particularly in pathological contexts such as the inflammatory bowel disease (IBD) and functional gastrointestinal disorders (FGIDs).

The first detection of P450scc and 3β‐HSD via in situ hybridization was observed in the mouse gut during the embryonic development.^37^ The production of PROG and androgens, such as DHEA and androstenedione, has also been identified in amphibians and rats.^9^, ^10^, ^11^ This capacity to metabolize sex steroids has been proposed as a mechanism that may influence colorectal cancer development.^12^ Specifically, in studies of colorectal cancer cell lines and normal gut mucosa, in vitro and in vivo, the expression of 17β‐HSD—an enzyme involved in androgen and estrogen metabolism—was observed.^38^, ^39^, ^40^ Furthermore, a detailed steroidogenic profile of PROG and T metabolites has been reported in colon of adult male naïve rats.^6^ In a comparative study among colon, plasma and cerebral cortex, it was demonstrated that local steroidogenesis of 3α, 5α‐metabolites of PROG and T was higher in the colon than in the cerebral cortex.^6^ In line with this, the expression of 3α‐HSOR gene was significantly higher in colon than in the cerebral cortex, suggesting a localized function for these metabolites.

The intestinal steroidogenesis has also been examined in the context of sexual dimorphism, where differences in the expression of factors between males and females are evident. The earliest evidence of sex‐dimorphic steroidogenic activity in the small intestine was observed in the frogs (*Rana esculenta*) via incubation with [4‐^14^C] cholesterol, showing higher PREG production in females.^8^ A similar result in adult naïve Sprague–Dawley rats, even after gonadectomy was reported.^14^ Specifically, the levels of gut steroids—such as PREG, PREG‐sulfate, PROG, DHP, ALLO, ISOALLO, DHEA, T, DHT, 3α‐diol, and 17β‐E—were quantified using liquid chromatography tandem mass spectrometry (LC–MS/MS) in both male and female rat colons, revealing several sexually dimorphic features of host metabolism.^14^

## 3 LOCAL STEROIDOGENESIS: BEHIND THE CONVENTIONAL ENDOCRINE REGULATION

The regulation of peripheral steroidogenesis in the adrenals and gonads is primarily governed by endocrine axes, such as the hypothalamic–pituitary–adrenal (HPA) axis and the hypothalamic–pituitary–gonadal (HPG) axis. However, these axes and their hormonal signals are not the only means of regulating local steroidogenesis. Other factors also contribute to this regulation.

For example, extra‐glandular steroidogenesis involves specific activators like the transcription factor liver receptor homolog‐1 (LRH‐1), which is closely related to the steroidogenic factor‐1 (SF‐1) and regulates steroidogenic enzymes in the adrenal glands.^35^ In the intestine, during an inflammatory event, glucocorticoids are essential for exerting anti‐inflammatory effects. Immune activation, potentially triggered by nuclear factor‐kappa B (NF‐kB) and tumor necrosis factor‐α (TNF‐α), enhances the gene expression of LRH‐1, thereby stimulating glucocorticoid synthesis.^41^, ^42^, ^43^ Indeed, overexpression of LRH‐1 has been shown to upregulate *Cyp11a1* and *Cyp11b1* gene expression in a murine intestinal epithelial cell line, leading to increase CORT production.^41^

While corticosteroid production in the adrenal glands is regulated by adrenocorticotropic hormone (ACTH), this mechanism is not as effective in the intestine, highlighting differential regulation between that adrenals and the gut.^35^, ^44^

In terms of local regulation and/or neuroendocrine control of sex steroid synthesis in the gut, evidence is still limited. However, the receptor for luteinizing hormone (LH), a key gonadotropin responsible for steroidogenesis in the ovaries and testis, is expressed in GIT of both humans and rats.^45^, ^46^, ^47^, ^48^ Although the presence of mRNA for neuroendocrine control molecules like gonadotropin‐releasing hormone (GnRH), ACTH and follicle‐stimulating hormone (FSH) remains undetectable or controversial in the gut,^44^, ^45^, ^46^, ^47^ these findings pave the way for future research using advanced techniques to better understand the neuroendocrine control of gut steroidogenesis.

## 4 THE GUT MICROBIOME AND STEROID MOLECULES

### 4.1 Microbial niches of gastrointestinal tract and gut steroidogenesis

The GIT consists of the esophagus, stomach, and intestines, which are further divided into the small and large intestines. Research has consistently demonstrated that different microbial communities and distinct immune cells thrive along various sections of the human GIT resulting in functional heterogeneity across segments.^49^ The abundance and diversity of microbes generally increase from the proximal to the distal portions of the intestine, with the colon hosting a highly active metabolic environment.^50^ Although the small intestine has a more rapid transit of food, recent reviews highlight an intimate interaction between the microbial community and the host's immune system, described as a “chit‐chatting” between the microbes and host immunity.^51^ These anatomical and functional aspects underline the importance of studying gut steroidogenesis as a potential mechanism of communication between the host and the gut microbiota, mediated by steroid signaling.

### 4.2 The gut microbiome as a virtual endocrine organ

The concept of the gut microbiome acting as a virtual endocrine organ, influencing distal organ function, is not actually a new aspect.^52^ Besides synthesizing important gut peptides and active neurotransmitters, the gut microbiota also possesses the metabolic ability to activate or deactivate sex steroids.^53^ The gut microbiome's biochemical potential is key to exploring the steroidome, the full set of steroids in cells or organisms. Steroid analysis has long been essential in diagnosing endocrine disorders, and advances in steroidomics now allow for comprehensive monitoring of steroids in biological samples. Notably, an important contribution of the human gut microbiota to host physiopathology is a functional estrobolome, which refers to a specific set of enteric bacterial genes whose products are capable of metabolizing estrogens.^54^ Especially important are bacterial species possessing β‐glucuronidase (GUS) and β‐glucuronides enzymes, which are involved in estrogen deconjugation and conjugation. For example, GUS enzymes can convert inactive estrogens back into their active forms, allowing them to re‐enter circulation.^55^ Although this activity is crucial for normal hormonal regulation, an excessive GUS activity can lead to higher levels of active estrogens, potentially contributing to hormone‐related disorders like cancer or endometriosis.^56^ These aspects are relevant also for androgen metabolism, given that GUS enzyme is involved in the deglucuronidation of DHT and T.^57^ Thus, its regulation by the gut microbiota is essential for maintaining hormonal balance.

However, mechanistic studies investigating steroid synthesis and metabolism by the gut microbiome are still ongoing, considering not only GUS enzymes but also other steroidogenic avenues, such as the ability to convert estrone into more potent estradiol.^58^ Another example are specific bacteria, such as *Steroidobacter denitrificans* ^59^ and *Comamonas testosteroni*, which can transform and utilize sex steroids.^60^ *Clostridium scindens*, a human gut microbe, has a high potential to convert glucocorticoids into androgens,^61^ while *Mycobacterium neoaurum* degrades T into androstenedione via 3β‐HSD, with its prevalence linked to depression in males.^62^ Additionally, in vitro studies have shown that specific bacterial strains can metabolize androgens, transforming T into DHT.^63^ For a detailed review of microbial steroid metabolism, please refer to references.^61^, ^64^, ^65^ Lastly, a new recent research has shown that certain organisms in the gut microbiota produce ALLO (a derivative of progesterone), converting the glucocorticoids found in human bile into progestins (via 21‐dehydroxylation).^66^ The ALLO levels were increased in feces from pregnant humans, suggesting a crucial role of specific gut microbes in the context of women's health and, implications in the post‐partum depression.

The composition of gut microbiota is closely associated with sex steroid levels, creating a reciprocal relationship. Indeed, among various factors influencing the gut microbiota, sex is a major contributor.^67^ Thus, if the microbiome is contributing to levels of steroid molecules, these in turn, may shape gut microbiota composition and function.^14^, ^68^, ^69^, ^70^, ^71^

### 4.3 Gut microbiota's role in modulating the steroid environment

The interaction between the metabolism of steroids regulated by gut microbiota and the microbiota itself plays a central role in maintaining host homeostasis. This interaction affects animals and humans under physiological and pathological conditions.^72^, ^73^ The human body exists in a symbiotic relationship with various microenvironments, both external and internal. The intestinal tract, in particular, is the most heavily colonized site, containing approximately 10^15^ microbial cells.^74^ These data highlight the critical role of the gut microbial community in human health, as microbes and their metabolites modulate intestinal homeostasis. For example, an intestinal metabolomic analysis revealed that treating mice with the antibiotic streptomycin affected the levels of over 87% of all detected metabolites, including steroids and eicosanoids, confirming the involvement of intestinal microbiota in these pathways.^75^ While strong correlations between specific taxa and steroid levels have been reported, fewer studies have explored the direct causal effects of the microbiome on steroid levels in host compartments. A number of studies, described below, report fecal microbial transplantation (FMT), in germ‐free (GF) and specific pathogen free (SPF) mice, highlighting a direct role of the microbiome in shaping the steroid environment. One interesting study showed that FMT in mice, from adult males to immature females, altered the recipient's microbiota, resulting in elevated T levels, metabolomic changes, and an amelioration of autoimmune disease progression.^76^ This is one of the few studies providing evidence for the crucial role of microbiota‐produced sex steroids and the potential therapeutic applications for FMT. Notably, the sexual dimorphism in metabolic complications caused by sex steroids remains an unexplored aspect of FMT. A cross‐sex FMT, proposed as a potential tool to address sex‐specific pathologies, highlights the importance of considering sex‐bias in such treatments.^77^ In both GF and SPF mice, the role of gut microbiome in de novo steroidogenesis was also demonstrated.^78^ Specifically, genes involved in the initial stages of steroidogenesis (e.g., *Stard3* and *Cyp11a1*) were upregulated in the small intestine and Peyer's patches in SPF mice, in response to acute immune stress, compared to GF mice. These findings confirmed local gut steroidogenesis and highlighted that, under stress conditions, the gut microbiota drives local CORT production.^79^, ^80^, ^81^ Future studies should focus on determining whether sex steroid production in the small and large intestine exhibits sexual dimorphisms in GF mice under physiological conditions. It would also be useful to evaluate how gut‐derived PROG metabolites, androgens and estrogens are modulated by the absence of gut microbiota and how this disruption impair communication with the brain.

## 5 GUT MICROBIOTA AND NEUROSTEROIDS IN CENTRAL NERVOUS SYSTEM

This specific scenario of gut microbiota, in both GF and SPF mice impacts neurosteroids (steroids synthesized in the brain).^82^, ^83^ Although these aspects were not studied in both sexes, several neurosteroid alterations were detected in male GF mice, indicating a strong association between gut microbiome and steroidogenesis in CNS. One key finding was that steroid pattern in plasma did not reflect changes in the brain.^82^ Specifically, two PROG metabolites (i.e., DHP and ISOALLO) showed a significant increase in the hippocampus, cerebral cortex and cerebellum, but not in plasma, suggesting a link between the microbiome and steroid pathway in the CNS, specifically on these active metabolites. In male GF mice compared to conventionalized mice, the absence of microbes also affected neuroactive steroids in plasma.^82^ For instance, a significant increase in ALLO and decrease in 3α‐diol, both important modulators of GABA‐A R, were detected. Interestingly, housing conditions also play a role—mice raised in general room (XZ) exhibited higher bacterial diversity compared to those housed in SPF environment. The difference let to various alterations in neurosteroid levels.^83^ For example, DHEA levels displayed a left‐lateralized pattern in the hippocampus and hypothalamus of SPF mice. These findings underscore that the absence of microbes or modifications in microbiota composition can impact neurosteroids in the brain, further supporting the idea of microbiota‐mediated modulation of neuroendocrine pathways critical to brain functions.

In the next section, we will discuss evidences showing how the intestinal steroid environment is modulated by pharmacological treatments, focusing on two examples: finasteride and selective‐serotonin reuptake inhibitors (SSRIs).

## 6 PHARMACOLOGICAL EFFECTS ON GUT STEROIDS AND MICROBIOTA

### 6.1 The case of finasteride

Finasteride (or Propecia) is a drug prescribed for androgenetic alopecia (AGA), a disorder characterized by high DHT levels that lead to miniaturization of the hair bulb and hair loss. The drug mechanism of action consists of the inhibition of the enzyme 5α‐R2, which mainly converts T into DHT in the skin. Apparently safe and effective, during the treatment, some patients experience fluctuations of mood, anxiety and depressive states associated with erectile dysfunction and low libido, which negatively affect their lives. Patients, who manifest persistent side effects after finasteride suspension are collectively named as post‐finasteride syndrome (PFS) patients.^84^, ^85^ A clinical study reported that in PFS patients, persistent alterations of steroid levels both in plasma and in cerebral spinal fluid were associated with andrological and psychiatric features.^86^ In addition, as demonstrated in the PFS animal model, this drug treatment and withdrawal induced altered levels of steroids not only in plasma and in cerebrospinal fluid but also in the brain.^87^ Interestingly, a 16S rRNA gene amplicon sequencing analysis of fecal samples from PFS patients detected a specific modification in gut microbiota community composition, suggesting a potential role in the etiopathology of this syndrome.^88^ Specifically, the α‐diversity was significantly lower in the PFS group versus healthy controls, suggesting a reduction in the richness of fecal samples with a specific taxonomic profile. In agreement, gut microbiota alterations have been reported in an experimental model of PFS.^89^ The effect of finasteride treatment and its withdrawal were also evaluated on the gut steroid levels.^90^ As previously reported, finasteride treatment not only decreased DHT levels as expected but also the levels of its active androgen metabolite 3α‐diol with a significant increase in T levels. Of note, a significant reduction in ALLO was observed in the colon of PFS rats, which persisted even after 1 month of withdrawal. In addition, the persistent decrease in ALLO levels was instead associated with an increase in PREG levels, suggesting a local relationship between these two steroid molecules.

Interestingly, the rescue of ALLO levels by subcutaneous injections with this steroid during the drug withdrawal ameliorated gut inflammation in the PFS animal model, suggesting an anti‐inflammatory effect of this progesterone metabolite.^90^ A putative mechanism of action of GABA‐A R subunits was proposed given that they are spread throughout the ENS^28^ and modulated at finasteride withdrawal, not only in the colon but also in the brain.^87^

Considering the important evidence suggesting the interaction of peroxisome proliferator‐activated receptor alpha (PPAR‐α) and ALLO in the GMBA,^91^ it will be crucial to investigate whether this interaction is altered in PFS animal model. Of note, PPAR‐α was proposed as a potential biomarker in IBD.^92^ Therefore, the potential significance of ALLO‐based intervention in shaping future treatment options could be definitely a new approach for PFS and IBD.

### 6.2 The selective‐serotonin reuptake inhibitors and gut steroids

Antidepressants are a heterogeneous group of drugs mainly prescribed for the treatment of psychiatric disorders, such as depression, obsessive‐compulsive disorder, panic attacks, anxiety, post‐traumatic stress disorder, premenstrual dysphoric disorder and menopause‐associated problems.^93^ All these disorders may potentially have psychiatric‐associated gastrointestinal problems and moreover, modifications of gut microbiota compositions, suggesting a strong interaction between gut‐microbiota and brain.

Furthermore, a subclass of antidepressants, the SSRIs, also have an off‐label use for the FGIDs. Indeed, a classic example of gut‐brain dysfunction in pathophysiology are the FGIDs, and the most globally prevalent is the irritable bowel syndrome (IBS). This disorder is more frequent in women^94^ and this sex bias was associated with sex steroids and the GMBA.^95^ The gastroenterologists diagnose and classify the patients with IBS according to the new Rome IV criteria, given that the symptoms can be highly diverse.^96^ The patients suffer of abdominal pain usually but not always, associated with symptoms including anxiety, depression, headache, and fatigue which seriously affect the quality of life.^97^ Despite its high global incidence, the mechanism of this disorder is still unclear and therapeutic options remain limited. Clinical trials have revealed important sex‐related differences in therapeutic efficacy of some modulators of serotonergic system used for IBS, suggesting a link between estrogens and serotonin in the modulation of stress‐related visceral hypersensitivity (VH).^98^, ^99^ Indeed, in preclinical studies, it has been shown that the visceral pain response varies across the estrous cycle^100^ and that interestingly, both estrous cycle and ovariectomy‐induced changes in visceral pain are microbiota‐dependent.^101^ In line with our results reporting a modulation of gut steroids after ovariectomy in rodents,^14^ it will be important to evaluate this scenario in IBS models.

The connection between FGIDs and SSRIs is particularly interesting because serotonin is mainly synthetized in the gut and plays a predominant role in regulation of motility and inflammation.^102^ However, perhaps less known, is the fact that SSRIs modulate both serotonin and steroid molecules, not only in the brain but also in the gut.^103^

Interestingly, a subchronic treatment with paroxetine, a SSRI, in naïve adult male rats alters the neurosteroids pattern.^104^ Specifically, higher ALLO levels were detected in the hippocampus and were associated with increased mRNA expression of the enzyme 3α‐HSOR. On the other hand, a completely different pattern was observed in the colon.^103^ In particular, albeit no differences in ALLO levels were observed, the relative 3α‐5α reduced metabolite of DHT (i.e., 3α‐diol) significantly decreased in accordance with low levels of 3α‐HSOR. The binding of the metabolites with non‐classical steroid receptor (GABA‐A receptor) both in the CNS and ENS, may suggest an opposite modulation of the GABAergic system, which cooperates in the communication of gut‐brain axis. In agreement, a significant decrease in α3, α4, δ subunits after paroxetine treatment was observed, suggesting that specific steroids impart changes in specific local neural circuits of the rodent colon.^103^ Of note, while plasma steroid levels remained unchanged, paroxetine treatment significantly modulated gut steroids. There was a notable decrease in androgen levels, with a corresponding increase in progesterone metabolites, particularly DHP and ISOALLO. Interestingly, in rodents a strong correlation between predicted microbial function (i.e., UDP‐N‐acetyl‐D‐glucosamine super‐pathway) and intestinal DHP was reported.^14^ In particular, this pathway is essential for microbe's cell wall metabolism and also used as a solubility promoter for the transport of conjugated sex steroids,^105^, ^106^ suggesting a putative new role of this steroid in host‐microbes communication.

Considering all evidence reported here, it could be reasonable to speculate that the SSRIs‐mediated beneficial effects in FGIDs, and more specifically in IBS, can be also mediated by a gut steroids‐microbiota cooperation. Therefore, addressing the influence of sex hormones and gut steroids as well, in the modulation of VH appears critical to develop novel therapeutic strategies.

## 7 PREGNENOLONE, VALUABLE STEROID IN THE PHYSIOPATHOLOGY OF BRAIN AND GUT

PREG is the first steroid formed from cholesterol via the mitochondrial P450scc enzyme and is further metabolized in the cytoplasm into key sex steroids and glucocorticoids (Figure 1). While less studied than its metabolites, PREG has independent signaling effects, albeit its mechanism remains unclear. In the brain, PREG inhibits tetrahydrocannabinol (THC) effects mediated by the cannabinoid receptor type 1 (CB1R), protecting against CB1R overactivation and cannabis intoxication.^107^ It also suppresses pro‐inflammatory cytokines, promoting neuroprotective and anti‐neuroinflammatory effects, particularly in the hippocampus, and enhances memory and cognition.^108^, ^109^, ^110^, ^111^, ^112^ A key distinction exists between PREG and PREG sulfate, the latter being as a modulator of N‐methyl‐D‐aspartate (NMDA) and neurotransmitter receptors.^113^

Post‐mortem studies have linked elevated PREG levels to schizophrenia and bipolar disorder,^114^ while depressed patients show lower cerebrospinal fluid PREG levels,^115^ suggesting a therapeutic role of neurosteroid PREG in CNS disorders.

In Parkinson's disease (PD), PREG reduces L‐DOPA‐induced dyskinesias by lowering striatal BDNF levels, offering a potential treatment for PD‐related motor symptoms.^116^

Additionally, PREG's metabolite, PROG, exhibits neuroprotective effects in the gut's myenteric plexus, aligning with findings in the brain.^117^, ^118^ PREG activates pregnane X receptor (PXR), particularly in the gut,^119^ promoting anti‐inflammatory responses and potentially playing a role in gastrointestinal and autoimmune disorders like type 1 diabetes (T1DM), where low PREG levels correlate with PXR dysfunction and cognitive impairment.^120^, ^121^, ^122^ Additionally, PREG levels were associated with high *Blautia*, a functional genus also found in T1DM patients.^123^

PREG's interaction with PXR and CB1R^24^ suggests its therapeutic potential in gastrointestinal diseases. Both receptors, PXR and CB1R are expressed in the colon, contribute to anti‐inflammatory responses,^124^, ^125^ and PXR activation alleviates inflammation in an IBD animal model by inhibition of NF‐kB signaling pathway.^120^ Sexual dimorphism in colonic PREG levels has been observed, with higher levels in females.^14^ Thus, PREG may be an interesting candidate to be further explored in sexually dimorphic pathologies where GMBA is affected, such as IBS and dysphoric premenstrual disorder. Notably, PREG increases after SSRI withdrawal,^103^ suggesting a compensatory anti‐inflammatory response in the colon that may counter post‐SSRI sexual dysfunction (PSSD). Changes in gut microbiota during paroxetine suspension further imply that PREG may play a role in mitigating pro‐inflammatory effects to cope with the side effects induced by paroxetine suspension.^103^

## 8 FUTURE PERSPECTIVES

While progress has been made in understanding the gut microbiome's role in the steroidogenesis and steroid metabolism, several critical gaps remain to be explored.

### 8.1 Microbiome influence on steroid biosynthesis

The specific microbial taxa and metabolic pathways involved in glucocorticoid and sex steroid synthesis are not fully understood. Future research should focus on identifying key microbial species and enzymes through techniques such as microbiome manipulation, metagenomics, and metabolomics to identify microbial genes and metabolites that directly participate in steroid biosynthesis.

### 8.2 Role of gut mucosa/ENS in steroidogenesis

The hypothesis that gut mucosa produces steroids via autocrine and paracrine mechanisms needs further validation. Studies should clarify whether these steroids act locally or on distant tissues through endocrine pathways. Investigating tissue‐specific gene expression and receptor localization could shed light on these mechanisms, mapping the distribution and action of steroids within the gut mucosa and beyond, as in the ENS. Furthermore, organoid models or gut mucosa cultures could be employed to assess steroid production and signaling in a controlled in vitro setting, providing valuable insights into the specific cellular targets involved.

### 8.3 Steroid signaling mechanisms

It is unclear whether glucocorticoids and sex steroids act through distinct autocrine/paracrine pathways or share common mechanisms. Comparative studies of their effects on various gut cell types—immune, epithelial, and neural—using techniques such as single‐cell RNA sequencing and spatial transcriptomics could be applied to dissect the cell‐type‐specific responses to steroid signaling within the gut, while CRISPR‐based gene editing could be used to modulate steroid receptors and assess the functional consequences on steroid signaling pathways.

### 8.4 Clinical implications of microbiome‐steroid interactions

The exact role of gut microbiome‐mediated steroid metabolism in disease pathogenesis remains poorly understood. Longitudinal human studies, combined with animal models, are needed to establish causal links between microbiome alterations and disease outcomes. Profiling the microbiome, steroid levels, and clinical phenotypes will help clarify these relationships.

## 9 CONCLUSIONS

In this review, we have addressed some aspects related to diabetes mellitus, FGIDs, IBD, IBS, PFS, and PSSD which involve steroid environment signaling throughout the GMBA. Moreover, we have highlighted the potential role of the intestinal steroidogenesis and therefore of gut steroids, which encompass glucocorticoids and sex steroid molecules in physiological and pathological conditions. The crucial role of gut microbiome in the steroid synthesis and metabolism is an intricate topic under investigation. Expanding the knowledge of microbial steroidome could be useful to evaluate the contribution of microbes in the regulation of steroid environment and in turn, how to shape microbiome for therapeutic strategies in which steroids can be affected.

Taken together, this review highlights new points of view considering steroids as potential therapeutic approach for gastrointestinal disorders and brain comorbidities.

## AUTHOR CONTRIBUTIONS

**Silvia Diviccaro:** Writing – original draft; writing – review and editing; conceptualization. **Silvia Giatti:** Writing – review and editing. **Lucia Cioffi:** Visualization. **Gabriela Chrostek:** Visualization. **Roberto Cosimo Melcangi:** Writing – review and editing.

## FUNDING INFORMATION

This work was supported by the DM 737/2021‐MUR and “Linea 3 My First SEED UNIMI.” This work was also supported by the MIUR Progetto Eccellenza.

## CONFLICT OF INTEREST STATEMENT

The authors declare no conflicts of interest.

## Data Availability

Data sharing not applicable to this article as no datasets were generated or analysed during the current study.
